# Amitriptyline’s anticholinergic adverse drug reactions–A systematic multiple-indication review and meta-analysis

**DOI:** 10.1371/journal.pone.0284168

**Published:** 2023-04-05

**Authors:** Maria-Sophie Brueckle, Elizabeth T. Thomas, Svenja Elisabeth Seide, Maximilian Pilz, Ana I. Gonzalez-Gonzalez, Truc Sophia Dinh, Ferdinand M. Gerlach, Sebastian Harder, Paul P. Glasziou, Christiane Muth

**Affiliations:** 1 Institute of General Practice, Goethe University, Frankfurt, Germany; 2 Centre for Evidence-Based Medicine, Nuffield Department of Primary Care Health Sciences, Radcliffe Observatory Quarter, University of Oxford, Oxford, United Kingdom; 3 Institute of Medical Biometry, University of Heidelberg, Heidelberg, Germany; 4 Institute of Clinical Pharmacology, Goethe University, Frankfurt, Germany; 5 Faculty of Health Sciences and Medicine, Bond University, Gold Coast, Australia; 6 Department of General Practice and Family Medicine, Bielefeld University, Bielefeld, Germany; NHRI: National Health Research Institutes, TAIWAN

## Abstract

**Background:**

Half the US population uses drugs with anticholinergic properties. Their potential harms may outweigh their benefits. Amitriptyline is among the most frequently prescribed anticholinergic medicinal products, is used for multiple indications, and rated as strongly anticholinergic. Our objective was to explore and quantify (anticholinergic) adverse drug reactions (ADRs) in patients taking amitriptyline vs. placebo in randomized controlled trials (RCTs) involving adults and healthy individuals.

**Methods:**

We searched electronic databases from their inception until 09/2022, and clinical trial registries from their inception until 09/2022. We also performed manual reference searches. Two independent reviewers selected RCTs with ≥100 participants of ≥18 years, that compared amitriptyline (taken orally) versus placebo for all indications. No language restrictions were applied. One reviewer extracted study data, ADRs, and assessed study quality, which two others verified. The primary outcome was frequency of anticholinergic ADRs as a binary outcome (absolute number of patients with/without anticholinergic ADRs) in amitriptyline vs. placebo groups.

**Results:**

Twenty-three RCTs (mean dosage 5mg to 300mg amitriptyline/day) and 4217 patients (mean age 40.3 years) were included. The most frequently reported anticholinergic ADRs were dry mouth, drowsiness, somnolence, sedation, fatigue, constitutional, and unspecific anticholinergic ADRs. Random-effects meta-analyses showed anticholinergic ADRs had a higher odd’s ratio for amitriptyline versus placebo (*OR* = 7.41; [95% CI, 4.54 to 12.12]). Non-anticholinergic ADRs were as frequent for amitriptyline as placebo. Meta-regression analysis showed anticholinergic ADRs were not dose-dependent.

**Discussion:**

The large OR in our analysis shows that ADRs indicative of anticholinergic activities can be attributed to amitriptyline. The low average age of participants in our study may limit the generalizability of the frequency of anticholinergic ADRs in older patients. A lack of dose-dependency may reflect limited reporting of the daily dosage when the ADRs occurred. The exclusion of small studies (<100 participants) decreased heterogeneity between studies, but may also have reduced our ability to detect rare events. Future studies should focus on older people, as they are more susceptible to anticholinergic ADRs.

**Registration:**

PROSPERO: CRD42020111970.

## Introduction

Approximately 51% of the general population use drugs with anticholinergic properties [1] and the percentage is rising [2]. Commonly observed adverse drug reactions (ADRs) associated with anticholinergic medicines such as amitriptyline are constipation, dry mouth, dry eyes, tachycardia, urinary retention, agitation, confusion, delirium, falls, hallucinations, and cognitive dysfunction [3]. In 2019, amitriptyline was prescribed more than eight million times in the USA, and listed as one of the hundred most commonly prescribed medicinal products [4]. A cross-sectional study based on a national sample of 2009–2010 Medicare Part D beneficiaries estimated that nearly one-third of nursing home residents in the USA used drugs with a high anticholinergic burden [5], and suffered from physical impairments and reduced ability to perform activities of daily living as a result [6]. Amitriptyline is used to treat major depressive disorder and other forms of depression, chronic pain, migraine, anxiety disorders [7], fibromyalgia [8], neuropathic pain [9], interstitial cystitis [10], nocturnal enuresis [11], eating disorders, and post-herpetic neuralgia [12].

ADRs associated with anticholinergic activity are underestimated and frequently overlooked in clinical management [3, 13]. They are often regarded as “unavoidable” and as part of the aging process or the course of a disease [14]. When misinterpreted as new symptoms of an existing disease, ADRs can lead to ‘prescribing cascades’ [15, 16], in which the drug reactions lead to the prescription of another medicinal product by the physician, or the increased use of over-the-counter products, rather than a discontinuation or dose adjustment of the responsible medicines [17]. ADRs have been defined as “an appreciable harmful or unpleasant reaction, resulting from an intervention related to the use of a medicinal product; ADRs usually predict hazard from future administration and warrant prevention, or specific treatment, or alteration of the dosage regimen, or withdrawal of the product.” [18]. With increasing age, reduced ability to metabolize drugs advances the risk of impairment associated with anticholinergic burden [19, 20].

Current research on anticholinergic effects is mostly based on observational data (e.g., [21, 22]), but such data can be biased because they do not distinguish ADRs associated with anticholinergic activity from disease symptoms and nocebo effects [23]. Evidence related to amitriptyline is generally focused on its effectiveness, benefits and harms with respect to a single indication (e.g. depression [7]). As ADRs are treatment‐specific rather than disease‐specific, our intention was to increase the number of ADRs available for analysis by combining the results of randomized controlled trials (RCTs) that compared treatment with amitriptyline and treatment with a placebo, regardless of indication and dose, and whether individuals were healthy or not. In this way, we hoped to provide a more comprehensive understanding of the harms of the medication. The objective of this multiple-indication systematic review and meta-analysis is thus to explore and quantify the frequency of ADRs associated with amitriptyline vs. placebo in randomized controlled trials (RCTs) of adults.

## Methods

This systematic review and meta-analysis was conducted in accordance with the Preferred Reporting System Items for Systematic Review and Meta-Analysis (PRISMA) checklist [24, 25]. It was conducted as part of EVITA (“Evidence-based multimedication program with implementation to practical care”; grant number: 01VSF16034), which aimed to update and upgrade the German guideline on polypharmacy [26]. The protocol was previously registered as PROSPERO CRD42020111970 and published in Systematic Reviews [27]. Each step was pilot-tested in order to train and calibrate the study team.

### Data sources and searches

The electronic databases MEDLINE, Embase, PsycINFO, PsycLIT, Psyndex, and the Cochrane Central Register of Controlled Trials were searched from inception, and free-text searches combined with controlled terms such as Placebo AND (Amitriptyline OR Amitriptylines OR Amineurin OR Amitrip OR Amitriptylin OR Amitrol OR Anapsique OR Damilen OR Domical OR Elavil OR Endep) AND Randomized controlled trials (for the complete search strategy see S1 File “Search Strategy”). We searched for RCTs from inception to September 2022. We performed citation analysis (forward and backward citation searches) on the studies included in Web of Science (including SCI—Science Citation Index Expanded, BIOSIS Citation Index, BIOSIS Previews, Current Contents Connect, Medline), and hand searched the reference lists of systematic reviews. To access the grey literature, we applied the methods proposed by AHRQ [28] that we describe in detail in our study protocol [27], and asked major amitriptyline manufacturers and experts about further relevant RCTs. Examples of the manufacturers we contacted include Sandoz, Neuraxpharm, and Hexal. From their inception until September 2022, we also searched the databases of the Food and Drug Administration (FDA), the European Medicines Agency (EMA), the clinical trial registries ClinicalTrials.gov, the International Standard Randomized Controlled Trial Number Register, and the WHO International Clinical Trials Registry Platform for unpublished studies.

### Study selection

Bibliographic details of all identified references were imported to Endnote and Covidence©, where they were independently screened (title, abstract, full text) for eligibility by two reviewers (MSB, ETT). We included randomized, double-blind, placebo-controlled trials (RCTs) on orally administered amitriptyline for any indication, dose and time period, as long as they included at least 100 adults (≥ 18 years) and reported quantitative data on ADRs group-wise. To avoid dissemination bias, we did not apply any restrictions to publication date or language. Full texts that were only available in languages other than English or German were translated by a native speaker. Any disagreement over eligibility was resolved through discussion or by a third reviewer (CM/PG).

### Data extraction and quality assessment

As recommended in the PRISMA statement [29], we developed a standardized data extraction sheet [27] from a set of variables defined a priori. We then pilot-tested the extraction sheet in a subsample of 20 studies to ensure inter-observer variation between the two reviewers was acceptably low. One investigator (MSB) extracted details on study design/setting, population, exposure, and outcomes of interest (e.g. all reported ADRs and adverse drug events such as falls), and two other investigators (ETT, MP) cross-checked the data. Conflicts were resolved by discussion or by another author (CM, SES). Efforts to obtain missing data from the authors of the included studies resulted in the addition of no further information. This was because the authors either no longer had access to study data [30, 31], or did not respond at all [32–36].

One investigator (MSB) conducted a quality assessment [37] of each study, while a second (ETT) verified the appraisal, and a third (AIGG) arbitrated in case of disagreement. To calculate the overall score, we used RoB 2, which is a revised tool for assessing risk of bias (RoB) in randomized trials [38]. For visualization we used the robvis web app [39].

### Data synthesis and analysis

Our primary outcome was the frequency of occurrence of ADRs that were indicative of anticholinergic activities (ACH-ADRs) as a binary outcome (absolute number of patients with/without any anticholinergic ADRs) in amitriptyline vs. placebo groups.

To ensure we had a good overview of existing data and could successfully recognize ADRs resulting from different signaling, we generated a classification scheme by extracting ADRs described for the general population in Martindale’s ’The Complete Drug Reference’ [40]. We then supplemented these with further reactions that Collamati et al. describe as being typical in an older population [1]. To prioritize the symptoms on the list, an experienced clinical pharmacologist (SH) first rated specificity for anticholinergic ADRs by differentiating symptoms that are unequivocally caused by the inhibition of muscarinergic signaling [41, 42] from those that are not. A detailed description of this process has been published in our study protocol [27].

In addition to the protocol, we analyzed ADRs that were not indicative of anticholinergic activity (NACH-ADRs) and general unspecific ADRs (G-ADRs) as primary outcomes. For studies that did not report the overall number of patients with/without ACH-ADRs/NACH-ADRs or G-ADRs, we selected the ACH-ADR/NACH-ADR or G-ADR that occurred most often in the respective study as primary outcome. Secondary outcomes included the frequency of individual ADRs and aggregated ADRs in the ACH-ADR, NACH-ADR and G-ADR categories. Individual ADRs were summarized to form aggregated ADR categories (most frequent individual ADR per study) and aggregated ADRs were summarized to create primary outcome categories (most frequent aggregated ADRs per study; see S1 Table: “Nesting of Outcomes”).

We supplemented the analysis with the risk difference (RD) and number needed to harm (NNH) to highlight the clinical implications of our results.

As all outcomes were binary, we used an odds ratio (OR) along with 95% confidence intervals (CI) in all analyses. For primary outcomes, we additionally employed risk difference (RD) along with 95% CIs. We provide a quantitative synthesis of findings from the included studies using the random-effects meta-analysis model with an inverse variance weighting and the DerSimonian-Laird estimator to assess heterogeneity between trials. To complement the analyses, a meta-regression was performed using dose as the independent continuous variable for each outcome.

We performed subgroup analysis for the following variables: sex (“male” vs. “female” when the majority of study participants were reported as such and “unknown” when no sex was reported in the study), mean daily dose (50–99mg, 100–150mg, >150mg), form of administration (capsules, tablets, other, unknown), and indication (depression, others). Unfortunately, and in contrast to the study protocol [27], the following variables lacked sufficient variation to enable subgroup analysis: duration of treatment, mean age, frailty, and multimorbidity. Sensitivity analyses were performed for low and medium (vs. high) RoB studies, as well as for studies with subjectively (vs. unknown) self-report outcomes. Initially, we also planned to distinguish between objectively measured and subjective self-reported outcomes, but fewer than five studies used objective measures, so no sensitivity analyses could be performed for this parameter. Forest plots were used for the visualization of study-specific results, and the combined effects of all meta-analyses [43]. We used funnel plots to assess evidence of publication bias, and Egger’s test to assess the skewness of the standardized deviates [44].

An analysis was only performed when at least 5 studies provided valid data, with the exception of funnel plots for which at least 10 studies were required for data to be considered valid. All analyses were performed in R version 3.6.1 or higher [45], using the extension meta (version 4.15–1) [46]. Even though the meta package provides results stemming from the use of fixed-effects models by default, we only used those from random-effects models. For the sake of completeness, the results from the use of fixed-effects models in the overall analysis can be found in Fig 2.

### Role of the funding source

The German Innovation Fund, which funded this review, was not involved in the design, conduct, analysis, or in drafting the manuscript.

## Results

**Fig 1:**
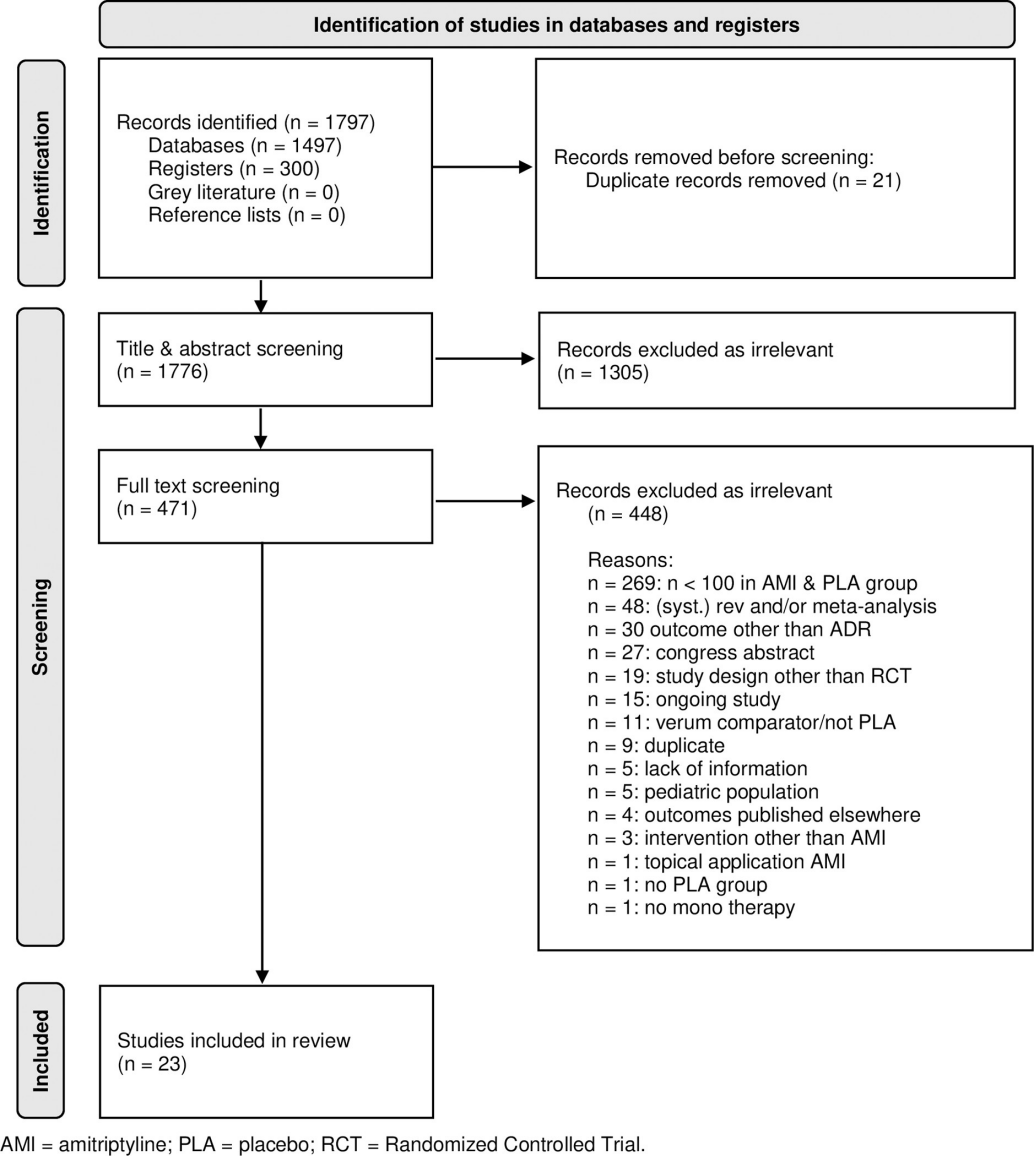
Flowchart of evidence selection based on PRISMA. AMI = amitriptyline; PLA = placebo; RCT = Randomized Controlled Trial.

Of the 1,898 studies imported for screening, 471 full texts were reviewed and 23 studies were eligible for data extraction and were included in the analysis (see Fig 1: “Flowchart of Evidence selection based on PRISMA”).

### Study characteristics

The included studies were mostly conducted in western countries and published between 1970 and 2018. Indications for the included populations were depressive disorders (n = 13), pain disorders (n = 9) and functional dyspepsia (n = 1).

Flexible dosing was used in 20 of the 23 studies (depending on the ADRs occurring in the individual) and the individual doses per day ranged from 5 to 300 mg across all studies. The study time (titration period) ranged from 1 week to 12 weeks with a median of 8 weeks. None of the studies specifically focused on older persons or patients with multimorbidity. None of the included studies reported adverse drug events (ADEs). Two of the included studies only reported overall ADRs [30, 36], and two other studies only reported overall ADRs and treatment discontinuation due to adverse effects [34, 47].

In total, 4217 patients of both sexes (67% female) with a mean age of 40.34 years participated in the 23 RCTs. Please see Table 1 “Study Characteristics” for more detailed information.

**Table 1:** Study characteristics.

ID	Indication	Total number of subjects	Age in years (both sexes)	Dosage amitriptyline per day (mg)	Accepted concomitant medications	Excluded concomitant medications	Dry mouth-related ADRs	Digestion-related ADRs	Genitourinary-related ADRs	Vision-related ADRs	Thermoregulation-related ADRs	Cardiovascular-related ADRs	Fatigue-related ADRs	Attention-related ADRs	Memory-related ADRs	Restlessness-related ADRs	Coordination-related ADRs	Unspecifically reported Ach-ADRs	Gastrointestinal-related ADRs	ADRs related to hypersensitivity	ADRs related to the endocrine system	Unspecifically reported NACH-ADRs	Unspecifically reported G-ADRs	ADRs overall	Discontinued due to ADRs
**Rickels 1970**	neurotic depression	136		100 (flexible dosing)			X						X			X				X					
**Feighner 1979**	depression	143		75–150 (flexible dosing)									X				X	X			X			X	X
**Goldberg 1980**	neurotic depression	122	18–60	75–200 (flexible dosing)																			X	X	X
**Rickels 1982**	depression	136		100–200 (flexible dosing)			X						X											X	
**Roffman 1982**	depression	214	18–65	75–150 (flexible dosing)	chloral hydrate		X	X		X			X			X						X			X
**Claghorn 1983**	major depression	172	18–65	75–300 (flexible dosing)	chloral hydrate		X	X	X	X		X	X	X		X	X		X			X	X	X	
**Amsterdam 1986**	depression/ anxiety	105	21–67	100–300 (flexible dosing)	chloral hydrate	sedative/hypnotic, anxiolytic medication																		X	X
**Reimherr 1990**	major depression	299	18–65	50–150 (flexible dosing)	chloral hydrate, given as infrequently as possible and not on nights before psychiatric scale ratings, as a sleeping aid; estrogens, progesterone, and diuretics	concurrent psychotherapeutic medication or concomitant medications, receiving another investigational drug within 4 weeks of enrolling in this study	X	X	X	X		X	X		X	X	X		X		X	X			X
**Carman 1991**	major depression	100	≥ 19	120–300 (flexible dosing)		contraception	X	X	X	X		X	X				X		X			X			X
**Bakish 1992**	major depression	112	18–65	50–150 (flexible dosing)	chloral hydrate, short-acting benzodiazepine	antihypertensive, diuretic, anticholinergic or sympathomimetic agents, psychotropic medication, foods rich in tyramine	X	X				X	X			X						X			X
**Pfaffenrath 1993**	tension type headache	131	18–65	25–75 (flexible dosing)		analgesics, mixed analgesics, ergotamine tartrate, dihydroergotamine, acetylsalicylic acid, paracetamol	X	X		X			X			X			X			X		X	X
**Carette 1994**	fibromyalgia	126	≥ 18	10–50 (flexible dosing)	acetaminophen	nonsteroidal anti-inflammatory drugs, hypnotic drugs, antidepressant agents																		X	X
**Bremner 1995**	major depression	100	≥ 18	40–280 (flexible dosing)	chloral hydrate		X	X		X		X	X			X	X		X	X	X	X			X
**Lydiard 1997**	major depression	260	≥ 18	50–150 (flexible dosing)	chloral hydrate, temazepam		X	X					X			X	X		X			X			X
**Montgomery 1998**	depression	386	≥ 18	40–280 (flexible dosing)	chloral hydrate	medication that might interfere with the action of mirtazapine, or the use of any psychotropic agent	X	X					X			X	X					X		X	
**Kautio 2009**	chemotherapy-induced neuropathic symptoms	114	20–75	25–100 (flexible dosing)		medication for neuropathic symptoms or contraindications for amitriptyline	X						X											X	
**Couch 2010**	migraine headache	391	18–70	25–100 (flexible dosing)				X	X	X	X	X	X			X	X		X	X		X	X		X
**Foster 2010**	interstitial cystitis/painful bladder syndrome	271	≥ 18	10–75 (flexible dosing)						X			X										X	X	X
**Goldman 2010**	arm pain associated with repetitive use	118	≥ 18	12.5–25 (flexible dosing)	anti-inflammatory medications, NSAIDs, antidepressants, other non-study treatments	starting new treatments during the study							X											X	X
**Dinat 2015**	HIV-associated sensory neuropathy	124	≥ 18	25–150 (flexible dosing)	acetaminophen, non-steroidal anti-inflammatory drugs, codeine phosphate		X						X										X		
**Talley 2015**	functional dyspepsia	194	18–75	25–50			X	X	X				X			X			X	X		X	X		
**Goncalves 2016**	migraine	131	18–65	25	acute migraine medication		X	X					X			X			X	X		X		X	
**Maarrawi 2018**	chronic neck pain	332	18–75	5								X	X			X									

Seventeen studies [10, 30, 32, 33, 36, 47–59] had a high, four studies [34, 60–62] a medium, and two studies [35, 63] a low overall RoB score. Fifteen of the seventeen studies with a high overall RoB score had an attrition rate of 20% or more. The results of the RoB assessment are shown in the S2 and S3 Tables.

### Primary outcomes

#### ADRs indicative for anticholinergic activity

**Fig 2:**
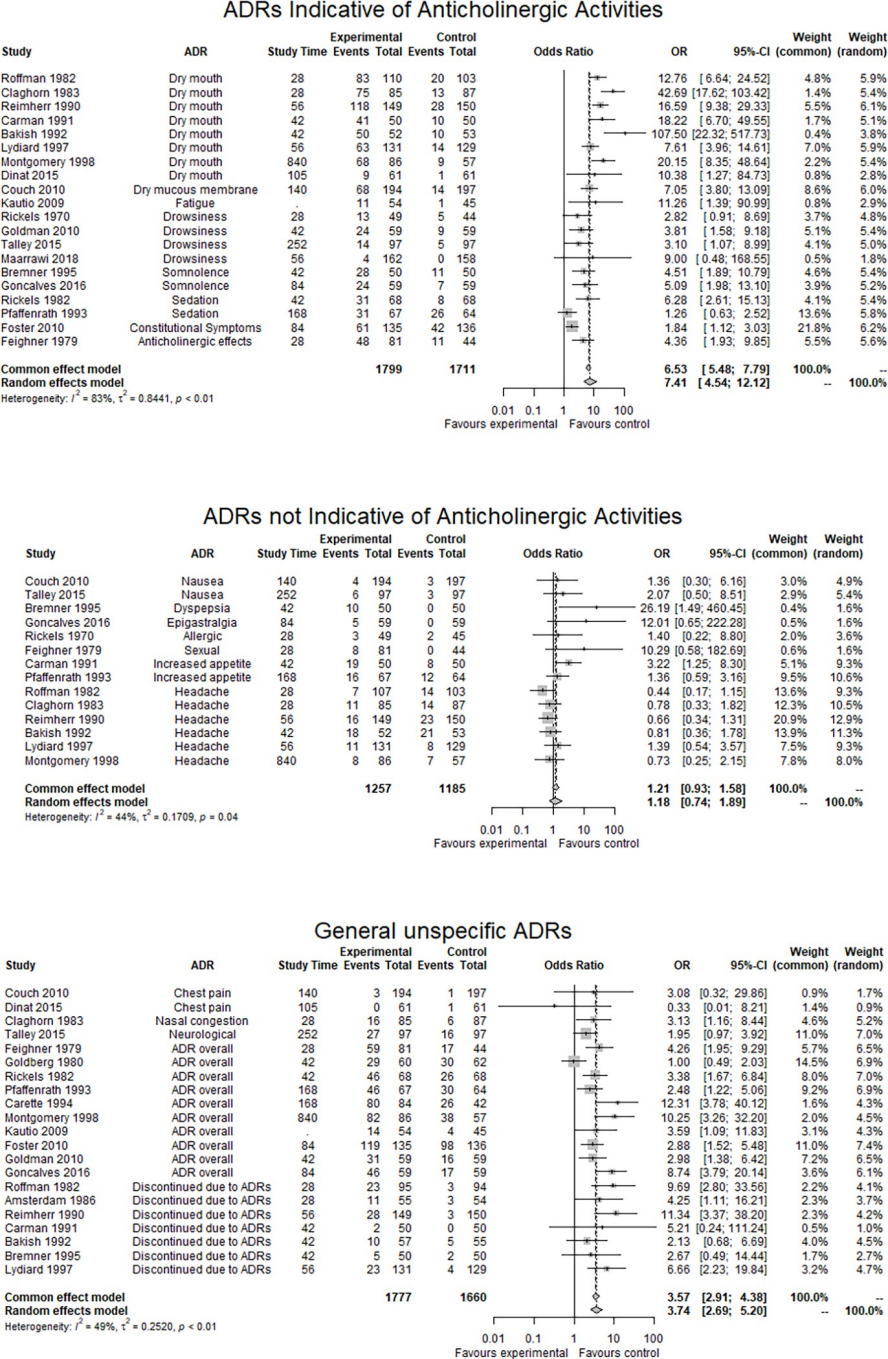
Forest plots of primary outcomes.

Twenty studies with a total of 3,510 participants were analyzed for ACH-ADRs. The most frequently reported ACH-ADRs were dry mouth (9 studies), drowsiness (4 studies), somnolence (2 studies), sedation (2 studies), fatigue (1 study), constitutional (1 study), and unspecifically reported ACH-ADRs (1 study). ACH-ADRs occurred significantly more often in the amitriptyline group than in the placebo group (*OR* = 7.41, [95% CI, 4.54 to 12.12], NNH = 2.89; *RD* = 0.35 [95% CI, 0.26 to 0.46]; see Fig 2 “Forest Plots of Primary Outcomes”) with a high observed heterogeneity in OR (*I*^2^ = 83%, *τ*^2^ = 0.84) and RD (*I*^2^ = 97%, *τ*^2^ = 0.05). The effects remained stable in sensitivity analyses involving only studies with low or medium RoB and studies with subjectively reported outcomes (see S4 Table). Adjusting for gender, indication, mean daily dose, and mode of administration did not substantially reduce heterogeneity (see S5 Table). Dose was not a predictor of the frequency of ACH-ADR (*B* = 0.01, *SE(B)* = 0.01, *p* = .40). In the assessment of publication bias, the funnel plot did not show significant asymmetry (*p* = .27).

#### ADRs not indicative for anticholinergic activity

Fourteen studies involving a total of 2442 participants were analyzed for NACH-ADRs. The most frequently reported NACH-ADR per study was headache (6 studies), nausea, increased appetite (2 studies each), dyspepsia, epigastralgia, allergic, and sexual ADRs (1 study each). NACH-ADRs did not occur significantly more often in the amitriptyline group than in the placebo group (*OR* = 1.18 [95% CI, 0.74 to 1.89], NNH = 34.27; *RD* = 0.03 [95% CI, -0.02 to 0.07]; see Fig 2 “Forest Plots of Primary Outcomes”) with moderate observed heterogeneity for OR (*I*^2^ = 44%, *τ*^2^ = 0.17) and RD (*I*^2^ = 61%, *τ*^2^ <0.01). Since only three studies had a low or medium RoB, no sensitivity analysis could be performed. Between-trial heterogeneity was lower when only studies with subjectively reported outcomes were taken into consideration (see S4 Table “Sensitivity Analyses”). Adjustment for gender, indication, mean daily dose, or mode of administration did not substantially reduce heterogeneity (see S5 Table “Subgroup Analyses of Primary Outcomes”). The frequency of NACH-ADR was not predicted by dose (*B* = 0.01, *SE(B)* = 0.01, *p* = .29). In assessing publication bias, the funnel plot showed significant asymmetry (*p* = .01).

#### General unspecific ADRs

Twenty-one studies involving a total of 3,437 participants were analyzed for G-ADRs. The most frequently reported G-ADRs per study were overall ADRs (10 studies), discontinuations due to ADRs (7 studies), chest pain (2 studies), nasal congestion, and neurological ADRs (1 study each). G-ADRs occurred significantly more often in the amitriptyline group than in the placebo group (*OR* = 3.74 [95% CI, 2.69 to 5.20], NNH = 6.23; *RD* = 0.16 [95% CI, 0.10 to 0.22]; see Fig 2 “Forest Plots”) with moderate observed heterogeneity of OR (*I*^2^ = 49%, *τ*^2^ = 0.25); and high heterogeneity of RD (*I*^2^ = 87%, *τ*^2^ = 0.01). The effects remained stable in sensitivity analyses that only involved studies with low or medium RoB, and studies including subjectively reported outcomes (see S4 Table “Sensitivity Analyses”). Adjustment for gender, indication, mean daily dose, or mode of administration did not substantially reduce heterogeneity (see S5 Table “Subgroup Analyses of Primary Outcomes”). The frequency of G-ADR was not predicted by dose (*B* = 0.01, *SE(B)* = 0.01, *p* = .54). The funnel plot did not show significant asymmetry when publication bias was assessed (*p* = .26).

#### Secondary outcomes

The results of the analysis of aggregated and individual ADRs were consistent with those of the main analysis, with ADRs, and especially those indicating anticholinergic activity, occurring more frequently in the amitriptyline group than in the placebo group (see Table 2 “Meta-analytical Results of Secondary Outcomes”).

**Table 2:** Meta-analytical results of secondary outcomes.

ADR	n_studies_	N_AMI_	N_PLA_	*OR*	95% CI	*I* ^2^	Favors*
***Aggregated ADRs***							
**ACH-ADRs**							
Dry mouth-related	15	1294	1247	11.10	(6.46; 19.06)	69%	PLA
Genitourinary-related	5	575	581	4.78	(1.57; 14.49)	0%	PLA
Coordination-related	8	826	764	4.43	(2,27; 8.36)	18%	PLA
Fatigue-related	20	1797	1709	3.94	(3.04; 5.11)	46%	PLA
Cardiovascular-related	7	742	745	3.06	(1.70; 5.51)	0%	PLA
Digestion-related	13	1262	1233	2.87	(2.12; 3.89)	16%	PLA
Vision-related	8	837	836	2.21	(1.06; 4.65)	50%	PLA
Restlessness-related	13	1288	1249	0.91	(0.53; 1.57)	39%	INC
**NACH-ADRs**							
Gastrointestinal-related	9	882	883	1.85	(0.73; 4.73)	61%	INC
Hypersensitivity-related	6	534	535	1.57	(0.46; 5.36)	0%	INC
Unspec. rep. NACH-ADRs	12	1127	1096	0.97	(0.67; 1.40)	18%	INC
**G-ADRs**							
Overall ADRs	11	808	690	3.85	(2.38; 6.24)	63%	PLA
Discontinued due to ADRs	13	1085	995	3.57	(2.26; 5.65)	13%	PLA
Unspec. rep. G-ADRs	5	572	578	1.65	(0.62; 4.37)	52%	INC
***Individual ADRs***							
**ACH-ADRs**							
Dry mouth	15	1100	1050	11.60	(6.42; 20.98)	70%	PLA
Somnolence	8	799	766	5.06	(4.01; 6.39)	0%	PLA
Tremor	8	826	764	4.43	(2.27; 8.63)	18%	PLA
Drowsiness	8	689	642	3.10	(1.96; 4.93)	9%	PLA
Constipation	13	1224	1194	3.06	(2.16; 4.34)	14%	PLA
Dizziness	10	1005	970	2.94	(1.91; 4.53)	25%	PLA
Fatigue	6	716	667	2.75	(1.67; 4.52)	0%	PLA
Insomnia	11	1189	1154	0.58	(0.39; 0.86)	0%	AMI
**NACH-ADRs**							
Nausea	6	706	710	1.21	(0.54; 2.71)	31%	INC
Headache	9	816	785	0.73	(0.55; 0.97)	0%	AMI

ADR = Adverse Drug Reaction; AMI = amitriptyline; PLA = placebo; OR = odds ratio; CI = confidence interval; ACH-ADRs = ADRs indicative of anticholinergic activity; NACH-ADRs = ADRs not indicative of anticholinergic activity; G-ADRs = general unspecific ADRs.

* PLA = more frequent ADRs in amitriptyline group (95% CI not including “1”); AMI = more frequent ADRs in placebo group (95% CI not including “1”); INC: inconclusive, i.e., no difference between placebo and amitriptyline regarding frequency of ADRs (95% CI includes “1”).

Nine aggregated ADRs occurred more frequently in the amitriptyline group, five were inconclusive, and none of them occurred more often in the placebo group.

Seven individual ADRs appeared more frequently in the amitriptyline group, one was inconclusive and two occurred more often in the placebo group (see Table 2 for more details).

## Discussion

To our knowledge, this is the first systematic review to compare ADRs associated with amitriptyline to placebo across all indications. Our results show that amitriptyline predominantly led to more frequent ADRs indicative of anticholinergic activity compared to placebo. Firstly, the odds of experiencing anticholinergic ADRs was about seven times higher overall. In keeping with the main analyses, the secondary analyses also showed a significant increase in ADRs related to dry mouth, genitourinary, coordination, fatigue, cardiovascular, digestion and vision symptoms with descending odds ratios declining from 11.1 to 2.21. The relatively high heterogeneity of 83% in the *I^2^* test in the primary analysis may be partly due to variation in the odds ratios of different combinations of anticholinergic ADRs in our primary outcome. Heterogeneity remained stable after adjustment for gender, indication, mean daily dose, and mode of administration. Secondly, the odds of experiencing general ADRs were four times higher in the amitriptyline than the placebo group, whereby we found no difference in ADR frequency for ADRs that are not indicative of anticholinergic activity. Sensitivity analyses showed the results to be robust, regardless of the RoB of the included studies and the methods applied in appraising ADRs.

Some of our results require explanation. Firstly, meta-analytic results of NACH-ADR included the symptom ‘headache’, for which the amitriptyline group performed better than the placebo group. This may have been due to amitriptyline´s indication as a prophylactic migraine treatment [64], which might have outweighed other NACH-ADRs. However, there were no significant differences between the amitriptyline and placebo groups for any of the secondary outcomes relating to gastrointestinal and hypersensitivity-related ADRs, as well as unspecific NACH-ADRs. Secondly, our results did not show any dose dependence of anticholinergic ADRs–neither in meta-regression nor in the subgroup analyses. Most of the included studies used their own individual titration methods (stopped titration or referred back to the last administered dose before ADRs occurred) and did not report the mean daily dose at the point of ADR occurrence, hence this result cannot be sufficiently substantiated.

A number of systematic reviews of the efficacy of amitriptyline vs. placebo have been conducted for specific indications and they have included ADRs as secondary outcomes [7–9]. Most concluded that the data was insufficient to analyze ADRs [8, 9]. Our multiple-indication review found a slightly higher risk of general ADRs than the reviews by Moore et al. [8, 9], but fewer risks than reported in the systematic review by Leucht et al. [7]. Leucht et al. reported an OR = 6.33 for anticholinergic ADRs in their meta-analysis on amitriptyline in depression, whereas we calculated an OR = 7.41. They reported higher ORs than we did for aggregated ADRs (for example genitourinary-related ADRs; OR: 8.73 vs. 4.78) and individual ADRs (for example dry mouth; OR: 13.50 vs. 11.60).

A major strength of our study is that we confined eligible studies to placebo-controlled RCTs. This is because disease symptoms and nocebo effects may bias observational studies [23], and verum comparisons in RCTs may be unhelpful because of the possible involvement of active substances that also have anticholinergic properties. However, two potential limitations should also be mentioned. First, the pre-defined inclusion criterion that RCTs require a sample size of at least 100 participants led to the exclusion of 269 small-scale studies, which potentially limited statistical power and our ability to detect rare events [65]. However, research has shown that ADR frequency estimates derived from very small trials (N<100) are highly unreliable [66], and that combining small-scale studies with large-scale studies can further increase heterogeneity between trials [67]. As a result, the inclusion of small-scale studies may actually make it more difficult to perform meta-analyses and hinder the detection of publication bias [68, 69]. The second limitation is that the average age of the study participants was very young (at around 40 years) limiting the generalizability of our results to older people. This is because older people are generally more sensitive to anticholinergic effects [70], and known to be at risk of certain harms, such as cognitive decline and falls [70, 71], which were not reported in the RCTs included in our review.

## Conclusion

Our multi-indication systematic review provides important evidence for clinical decision making. About one in three patients of about 40 years of age that are treated with amitriptyline will experience ADRs related to anticholinergic activity (RD = 0.35, NNH = 2.89). The potential to cause harm should be carefully weighed against potential benefits, and communicated to patients. Our results may even understate the situation in older individuals, who are generally more sensitive to anticholinergic effects [3]. Furthermore, the wide spectrum of anticholinergic symptoms supports individualized management, as patients may not be equally bothered by their symptoms. Furthermore, patient preferences should be taken into account, as patients differ in their desire for treatment to combat symptoms and negative outcomes. The paucity of studies examining more severe ADRs, such as cognitive decline and falls, may hinder the decision-making process and should be investigated in future studies. They should also seek to extend generalizability to include patients of older age.

## Supporting information

S1 Checklist PRISMA 2020. (DOCX)

S1 File Search strategy. (PDF)

S1 Table Nesting of outcomes. (PDF)

S2 Table RoB traffic light plot. (PDF)

S3 Table RoB summary plot. (PDF)

S4 Table Sensitivity analyses of primary outcomes. (PDF)

S5 Table Subgroup analyses of primary outcomes. (PDF)
