# Supplementary material for: Amitriptyline’s anticholinergic adverse drug reactions–A systematic multiple-indication review and meta-analysis
Source: PLoS One. 2023 Apr 5;18(4):e0284168. doi: 10.1371/journal.pone.0284168 (PMC10075391; doi:10.1371/journal.pone.0284168)
Supplement: S1 File — (PDF) [file pone.0284168.s002.pdf]

S2: Complete search strategy

PubMed

("Placebos"[Mesh] OR Placebos[tiab] OR Placebo[tiab] OR Placeboes[tiab] OR "Sham treatment"[tiab])

AND

("Amitriptyline"[Mesh] OR Amitriptyline[tiab] OR Amitriptylines[tiab] OR Amineurin[tiab] OR Amitrip[tiab] OR Amitriptylin[tiab] OR "Amitriptylin-neuraxpharm"[tiab] OR Amitriptylinneuraxpharm[tiab] OR Amitrol[tiab] OR Anapsique[tiab] OR "Apo-Amitriptyline"[tiab] OR ApoAmitriptyline[tiab] OR Damilen[tiab] OR Domical[tiab] OR Elavil[tiab] OR Endep[tiab] OR Laroxyl[tiab] OR Lentizol[tiab] OR Novoprotect[tiab] OR Saroten[tiab] OR Sarotex[tiab] OR Syneudon[tiab] OR Triptafen[tiab] OR Tryptizol[tiab] OR Tryptanol[tiab] OR Tryptine[tiab] OR Tryptomer[tiab] OR Amirol[tiab] OR Amit[tiab] OR Amitone[tiab] OR Amitor[tiab] OR Amitrol[tiab] OR Amrea[tiab] OR Amypres[tiab] OR Crypton[tiab] OR Eliwel[tiab] OR Enovil[tiab] OR Gentrip [tiab] OR Kamitrin[tiab] OR Latilin[tiab] OR Levate[tiab] OR Maxitrip[tiab] OR Mitryp[tiab] OR Odep[tiab] OR Redomex[tiab] OR Qualitriptine[tiab] OR Sarotena[tiab] OR Tadamit[tiab] OR Trepiline[tiab] OR Triad[tiab] OR Tripta[tiab] OR Triptaz[tiab] OR Triptyl[tiab])

AND

(Randomized controlled trial[pt] OR controlled clinical trial[pt] OR randomized[tiab] OR randomised[tiab] OR placebo[tiab] OR "drug therapy"[sh] OR randomly[tiab] OR trial[tiab]

OR groups[tiab])

NOT

(Animals[Mesh] not (Animals[Mesh] and Humans[Mesh]))

CENTRAL (Cochrane Library)

([mh Placebos] OR Placebos:ti,ab OR Placebo:ti,ab OR Placeboes:ti,ab OR "Sham treatment":ti,ab)

AND

([mh Amitriptyline] OR Amitriptyline:ti,ab OR Amitriptylines:ti,ab OR Amineurin:ti,ab OR Amitrip:ti,ab OR Amitriptylin:ti,ab OR Amitriptylin-neuraxpharm:ti,ab OR Amitriptylinneuraxpharm:ti,ab OR Amitrol:ti,ab OR Anapsique:ti,ab OR Apo-Amitriptyline:ti,ab OR ApoAmitriptyline:ti,ab OR Damilen:ti,ab OR Domical:ti,ab OR Elavil:ti,ab OR Endep:ti,ab OR Laroxyl:ti,ab OR Lentizol:ti,ab OR Novoprotect:ti,ab OR Saroten:ti,ab OR Sarotex:ti,ab OR Syneudon:ti,ab OR Triptafen:ti,ab OR Tryptizol:ti,ab OR Tryptanol:ti,ab OR Tryptine:ti,ab OR Tryptomer:ti,ab OR Amirol:ti,ab OR Amit:ti,ab OR Amitone:ti,ab OR Amitor:ti,ab OR Amitrol:ti,ab OR Amrea:ti,ab OR Amypres:ti,ab OR Crypton:ti,ab OR Eliwel:ti,ab OR Enovil:ti,ab OR Gentrip:ti,ab OR Kamitrin:ti,ab OR Latilin:ti,ab OR Levate:ti,ab OR Maxitrip:ti,ab OR Mitryp:ti,ab OR Odep:ti,ab OR Redomex:ti,ab OR Qualitriptine:ti,ab OR Sarotena:ti,ab OR Tadamit:ti,ab OR Trepiline:ti,ab OR Triad:ti,ab OR Tripta:ti,ab OR Triptaz:ti,ab OR Triptyl:ti,ab)

Embase (via Elsevier)

('placebo'/exp/mj OR Placebos:ti,ab OR Placebo:ti,ab OR Placeboes:ti,ab OR ‘Sham treatment’:ti,ab)

AND

('Amitriptyline'/exp/mj OR Amitriptyline:ti,ab OR Amitriptylines:ti,ab OR Amineurin:ti,ab OR Amitrip:ti,ab OR Amitriptylin:ti,ab OR Amitriptylin-neuraxpharm:ti,ab OR Amitriptylinneuraxpharm:ti,ab OR Amitrol:ti,ab OR Anapsique:ti,ab OR Apo-Amitriptyline:ti,ab OR ApoAmitriptyline:ti,ab OR Damilen:ti,ab OR Domical:ti,ab OR Elavil:ti,ab OR Endep:ti,ab OR Laroxyl:ti,ab OR Lentizol:ti,ab OR Novoprotect:ti,ab OR Saroten:ti,ab OR Sarotex:ti,ab OR Syneudon:ti,ab OR Triptafen:ti,ab OR Tryptizol:ti,ab OR Tryptanol:ti,ab OR Tryptine:ti,ab OR Tryptomer:ti,ab OR Amirol:ti,ab OR Amit:ti,ab OR Amitone:ti,ab OR Amitor:ti,ab OR Amitrol:ti,ab OR Amrea:ti,ab OR Amypres:ti,ab OR Crypton:ti,ab OR Eliwel:ti,ab OR Enovil:ti,ab OR Gentrip:ti,ab OR Kamitrin:ti,ab OR Latilin:ti,ab OR Levate:ti,ab OR Maxitrip:ti,ab OR Mitryp:ti,ab OR Odep:ti,ab OR Redomex:ti,ab OR Qualitriptine:ti,ab OR Sarotena:ti,ab OR Tadamit:ti,ab OR Trepiline:ti,ab OR Triad:ti,ab OR Tripta:ti,ab OR Triptaz:ti,ab OR Triptyl:ti,ab)

AND

(random* OR factorial OR crossover OR placebo OR blind OR blinded OR assign OR assigned OR allocate OR allocated OR 'crossover procedure'/exp OR 'double-blind procedure'/exp OR 'randomized controlled trial'/exp OR 'single-blind procedure'/exp NOT ('animal'/exp NOT ('animal'/exp AND 'human'/exp)))

NOT

('article in press'/it OR 'chapter'/it OR 'conference abstract'/it OR 'conference paper'/it OR 'editorial'/it OR 'erratum'/it OR 'letter'/it OR 'note'/it OR 'review'/it OR 'short survey'/it)

AND

([embase]/lim)

PsycINFO (via Ovid)

(exp Placebo/ OR Placebos.ti,ab. OR Placebo.ti,ab. OR Placeboes.ti,ab. OR Sham treatment.ti,ab.)

AND

(exp Amitriptyline/ OR Amitriptyline.ti,ab. OR Amitriptylines.ti,ab. OR Amineurin.ti,ab. OR Amitrip.ti,ab. OR Amitriptylin.ti,ab. OR Amitriptylin-neuraxpharm.ti,ab. OR Amitriptylinneuraxpharm.ti,ab. OR Amitrol.ti,ab. OR Anapsique.ti,ab. OR Apo-Amitriptyline.ti,ab. OR ApoAmitriptyline.ti,ab. OR Damilen.ti,ab. OR Domical.ti,ab. OR Elavil.ti,ab. OR Endep.ti,ab. OR Laroxyl.ti,ab. OR Lentizol.ti,ab. OR Novoprotect.ti,ab. OR Saroten.ti,ab. OR Sarotex.ti,ab. OR Syneudon.ti,ab. OR Triptafen.ti,ab. OR Tryptizol.ti,ab. OR Tryptanol.ti,ab. OR Tryptine.ti,ab. OR Tryptomer.ti,ab. OR Amirol.ti,ab. OR Amit.ti,ab. OR Amitone.ti,ab. OR Amitor.ti,ab. OR Amitrol.ti,ab. OR Amrea.ti,ab. OR Amypres.ti,ab. OR Crypton.ti,ab. OR Eliwel.ti,ab. OR Enovil.ti,ab. OR Gentrip.ti,ab.

OR Kamitrin.ti,ab. OR Latilin.ti,ab. OR Levate.ti,ab. OR Maxitrip.ti,ab. OR Mitryp.ti,ab. OR Odep.ti,ab. OR Redomex.ti,ab. OR Qualitriptine.ti,ab. OR Sarotena.ti,ab. OR Tadamit.ti,ab. OR Trepiline.ti,ab. OR Triad.ti,ab. OR Tripta.ti,ab. OR Triptaz.ti,ab. OR Triptyl.ti,ab.)

AND

(Randomized.ti,ab. OR randomised.ti,ab. OR placebo.ti,ab. OR drug therapy.mp. OR randomly.ti,ab. OR trial.ti,ab. OR groups.ti,ab.)

Clinicaltrials.gov

(Placebos OR Placebo)

AND

(Amitriptyline OR Amitriptylines OR Amineurin OR Amitrip OR Amitriptylin OR Amitrol OR Anapsique OR Damilen OR Domical OR Elavil OR Endep)

(Placebos OR Placebo)

AND

(Laroxyl OR Lentizol OR Novoprotect OR Saroten OR Sarotex OR Syneudon OR Triptafen OR Tryptizol OR Tryptanol OR Tryptine OR Tryptomer OR Amirol OR Amit)

(Placebos OR Placebo)

AND

(Amitone OR Amitor OR Amitrol OR Amrea OR Amypres OR Crypton OR Eliwel OR Enovil OR Gentrip OR Kamitrin OR Latilin OR Levate OR Maxitrip)

(Placebos OR Placebo)

AND

(Mitryp OR Odep OR Redomex OR Qualitriptine OR Sarotena OR Tadamit OR Trepiline OR Triad OR Tripta OR Triptaz OR Triptyl)

WHO ICTRP

Placebo AND Amitriptyline OR Placebo AND Amitriptylines OR Placebo AND Amineurin OR Placebo AND Amitrip OR Placebo AND Amitriptylin OR Placebo AND Amitrol OR Placebo AND Domical OR Placebo AND Elavil OR Placebo AND Endep OR Placebo AND Laroxyl OR Placebo AND Lentizol OR Placebo AND Novoprotect OR Placebo AND Sarotex OR Placebo AND Tryptine

ISRCTN Trial Registry

(Placebos OR Placebo)

AND

(Amitriptyline OR Amitriptylines OR Amineurin OR Amitrip OR Amitriptylin OR “Amitriptylin-neuraxpharm” OR Amitriptylinneuraxpharm OR Amitrol OR Anapsique OR “Apo-Amitriptyline” OR ApoAmitriptyline OR Damilen OR Domical OR Elavil OR Endep

OR Laroxyl OR Lentizol OR Novoprotect OR Saroten OR Sarotex OR Syneudon OR Triptafen OR Tryptizol OR Tryptanol OR Tryptine OR Tryptomer OR Amirol OR Amit OR Amitone OR Amitor OR Amitrol OR Amrea OR Amypres OR Crypton OR Eliwel OR Enovil OR Gentrip OR Kamitrin OR Latilin OR Levate OR Maxitrip OR Mitryp OR Odep OR Redomex OR Qualitriptine OR Sarotena OR Tadamit OR Trepiline OR Triad OR Tripta OR Triptaz OR Triptyl)
