# Supplementary material for: Amitriptyline’s anticholinergic adverse drug reactions–A systematic multiple-indication review and meta-analysis
Source: PLoS One. 2023 Apr 5;18(4):e0284168. doi: 10.1371/journal.pone.0284168 (PMC10075391; doi:10.1371/journal.pone.0284168)
Supplement: S1 Table — (PDF) [file pone.0284168.s003.pdf]

S3 Table: Nesting of Outcomes

Primary outcomes	Secondary outcomes	
	**Aggregated ADRs***	**Individual ADR***
ADRs indicative of anti-cholinergic activity (ACH-ADRs)	*Dry mouth-related ADRs* (15)	***Dry mouth*** (14)<br>**Dry mucous membrane** (1)
	Digestion-related ADRs (13)	*Constipation* (13)
	Genitourinary-related ADRs (5)	Urinary retention (2)<br>Difficulty urinating (1)<br>Micturition disorder (1)<br>Urogenital (1)
	Vision-related ADRs (8)	Blurred vision (4)<br>Amblyopia (2)<br>Vision abnormalities (2)
	Thermoregulation-related ADRs (1)	Sweat discoloration (1)
	Cardiovascular-related ADRs (7)	Tachycardia (3)<br>Palpitation (3)<br>Postural Hypotension (1)<br>Standing heartrate (1)<br>Supine heartrate (1)<br>Increased standing and supine pulse (2)
	Fatigue-related ADRs (20)	***Fatigue*** (6)<br>Asthenia (2)<br>***Drowsiness*** (8)<br>Dizziness (19)<br>Depression (2)<br>***Somnolence*** (8)<br>**Sedation** (4)<br>Sedative effects (drowsiness, sedation, excessive sleep) (1)<br>**Constitutional symptoms (primarily fatigue, malaise)** (1)
	Attention-related ADRs (1)	Confusion (1)
	Memory-related ADRs (1)	Amnesia (1)
	Restlessness-related ADRs (13)	Restlessness (1)<br>Agitation (3)<br>Nervousness (3)<br>Tingling (1)<br>Sensory system (1)<br>Anxiety (1)<br>Panic attack (1)<br>*Insomnia* (11)
	Coordination-related ADRs (8)	*Tremor* (8)<br>Hyperkinesia (1)<br>Hypokinesia (1)<br>Slurred speech (1)<br>Paresthesia (1)
	Unspecifically reported ACH-ADRs (1)	**Anticholinergic effects (dry mouth, constipation, visual disturbances)** (1)
ADRs not indicative of anticholinergic activity (NACH-ADRs)	*Gastrointestinal-related ADRs* (9)	***Nausea*** (6)<br>Vomiting (1)<br>**Dyspepsia** (4)<br>Bloating (1)<br>Diarrhea (3)<br>Hemorrhoids (1)<br>Intestinal fluid (1)<br>Metabolic nutritional (1)

		Anorexia (1)<br>Abdominal pain (2)<br>**Epigastralgia** (1)<br>Taste perversion (1)
	ADRs related to hypersensitivity (6)	Pruritus (2)<br>Rash (2)<br>**Allergic** (1)<br>Liver function abnormality (1)
	ADRs related to the endocrine system (3)	Impotence (1)<br>**Sexual** (2)
	Unspecifically reported NACH-ADRs (12)	Change in appetite (1)<br>**Increased appetite** (3)<br>Reduced appetite (1)<br>Sweat (3)<br>*Weight gain* (5)<br>***Headache*** (9)
General unspecific ADRs (G-ADRs)	*Unspecifically reported G-ADRs* (5)	**Chest pain** (2)<br>Heartburn (1)<br>Infection/fever (1)<br>Musculoskeletal (2)<br>**Nasal congestion** (1)<br>Otolaryngologic (1)<br>Pain (primarily headache) (1)<br>Respiratory (1)<br>Pulmonary (2)<br>Endocrine (1)<br>Hematologic (1)<br>Clostridium difficile infection (1)<br>**Neurological** (2)<br>Psychiatric (1)<br>Cardiovascular (1)<br>Dermatology/skin (2)<br>Renal/genitourinary (1)<br>Gynecologic (1)<br>Gastrointestinal (2)<br>Dreams (1)
	ADRs overall (11)	***ADRs overall***<br>(No specific ADRs reported) (11)
	Discontinued due to ADRs (13)	***Discontinued due to ADRs***<br>(No specific ADRs reported) (13)

bold = ADR included in primary outcome; italic = meta-analysis conducted (at least 5 studies); * = in brackets: number of included studies.
