# Supplementary material for: Amitriptyline’s anticholinergic adverse drug reactions–A systematic multiple-indication review and meta-analysis
Source: PLoS One. 2023 Apr 5;18(4):e0284168. doi: 10.1371/journal.pone.0284168 (PMC10075391; doi:10.1371/journal.pone.0284168)
Supplement: S2 Table — (PDF) [file pone.0284168.s004.pdf]

S4 Table: RoB Traffic Light Plot

Risk of bias domains

Study	D1	D2	D3	D4	D5	Overall
amsterdam_1986	-	+	+	X	+	X
bakish_1992a	-	+	-	X	+	X
bremner_1995	-	-	-	+	+	X
carette_1994	+	-	-	+	+	-
carman_1991	-	+	+	+	+	-
claghorn_1983b	-	+	+	X	+	X
couch_2010	+	+	+	X	+	X
dinat_2015	+	+	+	+	+	+
feighner_1979	-	+	+	+	+	-
foster_2010	-	+	+	X	+	X
goldberg_1980	+	+	+	X	+	X
goldman_2010	+	+	+	+	+	+
goncalves_2016	-	+	+	X	+	X
kautio_2009	-	+	+	X	+	X
lydiard_1997	-	-	-	+	+	X
maarrawi_2018	+	+	+	X	+	X
montgomery_1998	-	+	+	X	+	X
pfaffenrath_1993	-	+	+	+	+	-
reimherr_1990	-	+	+	X	+	X
rickels_1970	-	-	-	+	+	X
rickels_1982	-	+	+	X	+	X
roffman_1982	-	+	+	X	+	X
talley_2015	+	+	+	X	+	X

Domains:
D1: Bias due to randomisation.
D2: Bias due to deviations from intended intervention.
D3: Bias due to missing data.
D4: Bias due to outcome measurement.
D5: Bias due to selection of reported result.

Judgement
X High
- Some concerns
+ Low
