# Supplementary figures and images for: Amitriptyline’s anticholinergic adverse drug reactions–A systematic multiple-indication review and meta-analysis
Source: PLoS One. 2023 Apr 5;18(4):e0284168. doi: 10.1371/journal.pone.0284168 (PMC10075391; doi:10.1371/journal.pone.0284168)

S5 Table: RoB Summary Plot

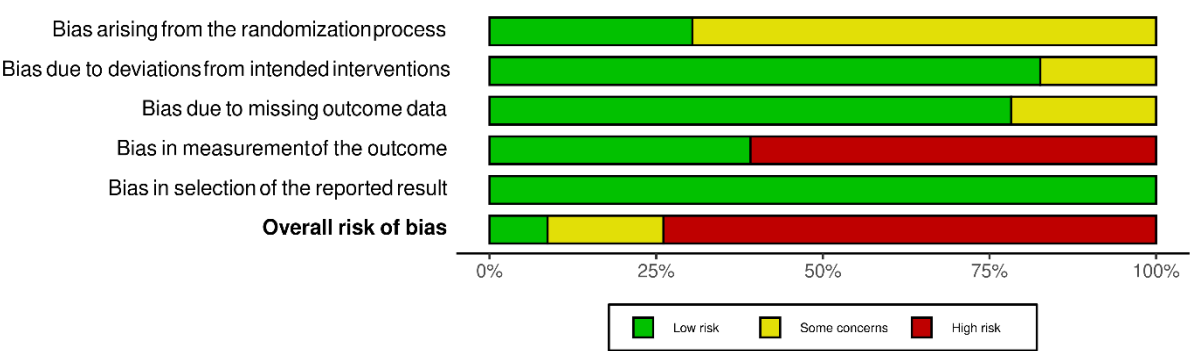

Supplement: S3 Table — (PDF) [file pone.0284168.s005.pdf]
