# Supplementary material for: Amitriptyline’s anticholinergic adverse drug reactions–A systematic multiple-indication review and meta-analysis
Source: PLoS One. 2023 Apr 5;18(4):e0284168. doi: 10.1371/journal.pone.0284168 (PMC10075391; doi:10.1371/journal.pone.0284168)
Supplement: S4 Table — (PDF) [file pone.0284168.s006.pdf]

S6 Table: Sensitivity Analyses of Primary Outcomes

Sensitivity Analysis	$n_{studies}$	N_{AMI}	N_{PLA}	*OR*	95% CI	I^2	τ^2	Favors*
ACH-ADRs								
Low or medium RoB	5	318	278	4.70	(1.29; 17.17)	80%	.87	PLA
Subjective outcome	8	561	505	4.05	(2.26; 7.26)	58%	.29	PLA
NACH-ADRs								
Subjective outcome	5	387	341	1.62	(0.74; 3.52)	0%	<.01	INC
G-ADRs								
Low or medium RoB	6	402	320	3.66	(1.75; 7.65)	36%	.10	PLA
Subjective outcome	6	445	397	4.54	(2.44; 8.46)	26%	.04	PLA

AMI = amitriptyline; PLA = placebo; OR = odds ratio; CI = confidence interval; I^2 = heterogeneity; τ^2 = between-study variance; RoB = risk of bias; ACH-ADRs = ADRs indicative of anticholinergic activity; NACH-ADRs = ADRs not indicative of anticholinergic activity; G-ADRs = general, unspecific ADRs.

*PLA = more frequent ADRs in amitriptyline group (95% CI not including "1"); AMI = more frequent ADRs in placebo group (95% CI not including "1"); INC: inconclusive, i.e., no difference in frequency of ADRs between placebo and amitriptyline groups (95% CI includes "1").
