# Supplementary material for: Amitriptyline’s anticholinergic adverse drug reactions–A systematic multiple-indication review and meta-analysis
Source: PLoS One. 2023 Apr 5;18(4):e0284168. doi: 10.1371/journal.pone.0284168 (PMC10075391; doi:10.1371/journal.pone.0284168)
Supplement: S5 Table — (PDF) [file pone.0284168.s007.pdf]

S7 Table: Subgroup Analyses of Primary Outcomes

Subgroup Analysis	$n_{studies}$	N_{AMI}	N_{PLA}	*OR*	95% CI*	I^2	τ^2	Favors**
ACH-ADRs	**20**	**1799**	**1711**	**7.41**	**(4.54; 12.12)**	**83%**	**0.84**	**PLA**
**Gender**								
Female	3	221	215	17.14	(0.41; 719.77)	81%	1.80	INC
Male	15	1397	1317	5.95	(3.44; 10.29)	83%	0.80	PLA
Unknown	2	181	179	10.81	(0.05; 2488.17)	51%	0.84	INC
**Indication**								
Depression	11	911	835	11.59	(5.93; 22.64)	75%	0.67	PLA
Other	9	888	876	3.58	(2.03; 6.32)	64%	0.34	PLA
**Mode of administration**								
Capsules	11	794	745	11.04	(5.50; 22.18)	74%	0.74	PLA
Tablets	5	565	524	4.11	(1.39; 12.14)	73%	0.57	PLA
Others	2	232	233	2.03	(0.16; 25.57)	0%	0	INC
Unknown	2	208	209	3.70	(0.01; 1.69e+04)	77%	0.54	INC
**Mean daily dose**								
<100mg	10	974	933	4.59	(2.41; 8.75)	76%	0.62	PLA
>=100mg & <=150mg	7	627	622	16.04	(6.55; 39.27)	74%	0.62	PLA
>150mg	3	198	156	4.52	(1.83; 11.19)	0%	0	PLA
NACH-ADRs	**14**	**1257**	**1185**	**1.18**	**(0.74; 1.89)**	**44%**	**0.18**	**INC**
**Gender**								
Female	3	218	215	0.73	(0.08; 6.78)	57%	0.07	INC
Male	9	858	791	1.05	(0.61; 1.78)	29%	0.02	INC
Unknown	2	181	179	2.11	(0.01; 444.01)	34%	0.12	INC
**Indication**								
Depression	10	840	768	1.07	(0,57; 2.01)	53%	0.23	INC
Other	4	417	417	1.64	(0.69; 3.93)	0%	<.01	INC
**Mode of administration**								
Capsules	8	610	574	1.11	(0.54; 2.30)	54%	0.26	INC
Tablets	3	342	305	1.54	(0.35; 6.75)	0%	< .01	INC
Unknown	2	208	209	0.97	(0.00; 1.06e+08)	72%	3.02	INC
Others	1	97	97	2.07	(0.50; 8.51)	NA	NA	INC
**Mean daily dose**								
<100mg	5	503	474	1.33	(0.63; 2.82)	0%	<.01	INC
>=100mg & <=150mg	7	624	622	1.06	(0.45; 2.48)	63%	0.32	INC
>150mg	2	130	89	2.76	(0.00; 4.46e+05)	24%	0.47	INC
G-ADRs	**21**	**1777**	**1660**	**3.74**	**(2.69; 5.20)**	**49%**	**0.25**	**PLA**
**Gender**								
Female	4	266	262	5.51	(1.79; 16.98)	37%	0.20	PLA
Male	15	1330	1219	3.27	(2.19; 4.88)	51%	0.23	PLA
Unknown	2	181	179	6.47	(2.41; 17.38)	0%	0.25	PLA
**Indication**								
Depression	12	967	900	4.02	(2.48; 6.50)	54%	0.34	PLA
Other	9	810	760	3.47	(2.02; 5.96)	47%	0.19	PLA
**Mode of administration**								
Capsules	12	850	810	3.48	(2.24; 5.40)	45%	0.25	PLA
Tablets	5	487	408	3.86	(1.27; 11.67)	47%	0.26	PLA

Others	2	232	233	2.41	(0.20; 28.48)	0%	0	INC
Unknown	2	208	209	9.50	(2.03; 44.43)	49%	0.25	PLA
**Mean daily dose**								
<100mg	11	896	817	3.84	(2.24; 6.58)	51%	0.24	PLA
>=100mg & <=150mg	8	677	677	3.70	(1.75; 7,81)	65%	0.55	PLA
>150mg	2	204	166	3.81	(2.68; 5.42)	0%	0	PLA

AMI = amitriptyline; PLA = placebo; OR = odds ratio; CI = confidence interval; I^2 = heterogeneity; τ^2 = between study variance; ACH-ADRs = ADRs indicative for anticholinergic activities; NACH-ADRs = ADRs not indicative for anticholinergic activities; G-ADRs = general unspecific ADRs.

*using scientific notation for values greater than 9,999. **PLA = more frequent ADRs in amitriptyline group (95% CI not including "1"); AMI = more frequent ADRs in placebo group (95% CI not including "1"); INC: inconclusive, i.e., no difference in frequency of ADRs between placebo and amitriptyline groups (95% CI includes "1"). ***Sex ("male" vs. "female", when the majority of study participants were reported as such, and "unknown" when no sex was reported in the study)
